# A244 SIMILAR RISK OF INCIDENT HEPATOCELLULAR CARCINOMA IN IMMIGRANTS/REFUGEES WITH HEPATITIS B CIRRHOSIS REGARDLESS OF THEIR REGION OF ORIGIN

**DOI:** 10.1093/jcag/gwac036.244

**Published:** 2023-03-07

**Authors:** P Wang, M Djerboua, J Flemming

**Affiliations:** 1 Gastroenterology; 2 ICES, Queen's University, Kingston, Canada

## Abstract

**Background:**

In Canada, over 75% of new immigrants/refugees come from HBV-endemic areas. Whether the risk of hepatocellular carcinoma (HCC) in immigrants/refugees with HBV cirrhosis is associated with their region of origin has not been reported.

**Purpose:**

The aim of our study was to describe the epidemiology of HCC in foreign-born individuals with HBV cirrhosis stratified by region of origin.

**Method:**

Population-based retrospective cohort study using administrative healthcare data in Ontario, Canada. Recent immigrants/refugees with HBV (sAg+ or DNA+) and cirrhosis from 2000–2017 were identified and stratified by World Bank region of origin by linkage to the Immigration, Refugees and Citizenship Canada Permanent Resident Database. Individuals were followed until HCC, death, liver transplant, or end of study (2020). The 1-, 5-, and 10-year cumulative incidences (CI) of HCC were calculated. Using adjusted competing risks regression, the subdistribution hazard ratios (sHR) for HCC were calculated with region of origin as the main exposure where death/LT were competing events.

**Result(s):**

4,438 immigrants/refugees were included (64% male, median age 47 years [IQR 37–56], 72% Asia/Pacific, 9% South Asia, 6% Sub-Saharan Africa, 3% Latin America/Caribbean, 7% Europe/Central Asia, 3% Middle East/North Africa) with 430 (10%) developing incident HCC over a median of 7.9 years (IQR 4.5-11.7). Age at HCC diagnosis was lowest in those from Sub-Saharan Africa (53 years IQR [44-58]). Overall, the annual incidence of HCC was 1.14/100 person-years. The CI of HCC at 1-, 5-, and 10 years was highest in those from Latin American/Caribbean (6.8%, 10.3%, and 13.1%), East Asia/Pacific (4.5%, 7.7%, 10.3%) and South Asia (4.0%, 7.2%, 10.3%). After adjusted competing risk regression (Table), decompensated cirrhosis (sHR 2.5; 95% CI 1.7–3.5), male sex (sHR 2.2; 95% CI 1.7–2.7), and age (sHR 1.05, 95% CI 1.04–1.06) were independently associated with HCC, while region of origin was not.

**Conclusion(s):**

Among immigrants/refugees with HBV cirrhosis, HCC is associated with male sex, older age, and decompensated cirrhosis but not with their region of origin. Efforts ensuring timely detection and evaluation of HBV in immigrants/refugees with a goal to prevent development of decompensated cirrhosis and provide early detection of HCC should be aggressively pursued regardless of their region of origin.

**Please acknowledge all funding agencies by checking the applicable boxes below:**

None

**Disclosure of Interest:**

None Declared

